# Common challenges faced by early-career researchers in Latin American and small U.S. universities

**DOI:** 10.1128/mbio.00238-25

**Published:** 2025-05-13

**Authors:** Daniela Cejas, Fatima Rodriguez Acosta, Juan Rivera-Correa

**Affiliations:** 1 Universidad de Buenos Aires, Facultad de Farmacia y Bioquímica 62963 , Buenos Aires, Argentina; 2 Consejo Nacional de Investigaciones Cientíﬁcas y Técnicas (CONICET) 62873, Buenos Aires, Argentina; 3 Departamento de Microbiología, Instituto de Investigaciones en Ciencias de la Salud, Universidad Nacional de Asunción 187173, San Lorenzo, Paraguay; 4 Biological Sciences Department, New York City College of Technology, City University of New York 2009, New York, New York, USA; 5 Biology PhD Program, CUNY Graduate Center 14772, New York, New York, USA; Instituto Carlos Chagas, Curitiba, Brazil

**Keywords:** Latin America, small U.S. universities, early-career researchers

## Abstract

Early-career researchers from Spanish-speaking Latin American countries and small U.S. universities are underrepresented in international scientific databases. They have geographical importance, infectious disease endemicity, and exceptional researchers in microbiology, but these factors do not translate to representation in high-impact scientific publications. Many reasons could be involved, including financial burdens such as the inability to pay article processing charges. Additional teaching, institutional, and service responsibilities also highly influence their research productivity. Despite this, they are expected to publish high-impact articles, and their career development highly depends on it. There is an opportunity for global peer collaboration to tackle this inequity and uplift underrepresented scientists, which will ultimately provide benefits and sustainability to the global microbial sciences.

## EDITORIAL

The Spanish-speaking Latin America region (LATAM) entails low representation in international databases (1) despite exhibiting incredible microbial diversity and endemicity for numerous infectious agents that have been tackled by skilled local microbiologists. Many factors, such as the inability to pay article processing charges (APCs), force LATAM researchers to publish in low-impact national journals or not publish at all. Their publications represent less than 1% of articles in gold (charge an APC) and 25% in diamond open-access journals (no APC charge) (1), 2.64% of the yearly total biomedical and life science publications (2), and have fewer citations even when published in prestigious journals (3, 4). Some of these constraints, such as limited funding and a significant gap in the number of publications compared to research-intensive institutions, are shared with small U.S. universities (5). This disparity is evident with studies reporting that the top 25% of institutions produce 89% of scientific articles from the United States (6). Ironically, both LATAM and small U.S. universities share the strict requirement of high-impact publications for promotion.

Scientists everywhere have a high workload connected to standard research commitments. LATAM researchers, similarly to small U.S. universities (7), have additional heavy teaching workloads (8). In many instances, teaching activities represent the main justification for their salary due to their limited grant funding (9, 10), which is highly connected to the country’s and institution’s investment in research and development (11). These researchers also face excessive institutional administrative responsibilities and run specialized clinical laboratory services for the public, which further dampens their research efforts. The reduction in research productivity by these additional duties, conversely, lowers their competitiveness for national and international funding opportunities, feeding into this cycle and affecting their academic career development (12).

Publications are a major component of science. Researchers are under strong pressure to publish their research to obtain career promotions, project funding, and economic benefits (and usually salary) (13). Academic evaluations will harshly measure both the quantity and where the researchers have published their results. A special emphasis and prestige are put on the first quartile (Q1) journals, which decline moving down through the quartiles. Most Q1 journals tend to be open access, charging expensive APCs, while the ones that do not are extra competitive due to the large number of submissions. For example, an average Q1/Q2 journal APC is over $2,000 (14), which can be the total or a significant amount of the total project funding for LATAM and small U.S. universities (15, 16). These costs are hard to bear by LATAM and small U.S. universities, and they opt to publish their research in cheaper, low-impact factor/Q journals, regardless of their work quality, which affects their impact (1). These issues largely affect ECRs whose permanent position depends on publications in high-impact factor/Q journals, even though they face the inability to pay APCs (17, 18).

International societies and open-access publishing companies are in a privileged position to tackle this inequity. One possible remedy is to modify publication fees not only depending on the country of origin or society membership but also considering the local funding (e.g., charging a proportion of the funds obtained). Another possibility is the opportunity to get substantial waivers by participating as volunteers in special programs (e.g., supporting network to help prepare and revise manuscripts). Moreover, Q1 journals could make specific research topic calls for international research in underrepresented sites, particularly for scientists working at infection-endemic locations, which would be a targeted way to tackle these issues. Addressing this issue and uplifting LATAM and small U.S. university researchers will provide sustainable and long-standing advancements for the global microbial sciences.
